# Routine Group-and-Save (G+S) Testing Prior to Laparoscopic Appendectomy: A 10-Year Single-Centre Review

**DOI:** 10.7759/cureus.99234

**Published:** 2025-12-14

**Authors:** Oday Al-Asadi, Mostafa Mahran, Roham Karimi, Karim Ataya, Farah Aldhaher, Mohammad Yousef

**Affiliations:** 1 General Surgery, Homerton University Hospital, London, GBR; 2 Upper Gastrointestinal (GI) Surgery, Homerton University Hospital, London, GBR; 3 Esophagogastric Surgery, Salford Royal NHS Foundation Trust, London, GBR; 4 Radiology, Guy's and St Thomas' NHS Foundation Trust, London, GBR

**Keywords:** bleeding and blood products, cost reduction, group-and-save testing, healthcare trust, laparoscopic emergency surgery

## Abstract

Background: Routine preoperative group-and-save (G+S) testing remains common before laparoscopic appendectomy in UK hospitals, despite limited evidence of its necessity. This study evaluated the clinical need and cost-effectiveness of routine G+S sampling in patients undergoing laparoscopic appendectomy at a single centre over 10 years.

Methods: We conducted a retrospective cohort study at Homerton University Hospital, London, UK, including all patients who underwent emergency or elective laparoscopic appendectomy between October 2014 and October 2024. Electronic records were used to identify patients and any perioperative transfusions. For transfused patients, operative and medical records were reviewed to determine the indication and timing of transfusion (surgical bleeding vs. other causes, intraoperative vs. postoperative). Demographic and clinical data were extracted by two independent reviewers and anonymized. We compared our transfusion rate to those reported in six published studies using two-proportion z-tests (significance p<0.05). A cost analysis estimated the financial impact of routine G+S, using the National Health Service (NHS) cost of £15-£21 per test.

Results: Among 1,733 patients (mean age 51 years; 925 males, 808 females), five (0.29%) received blood transfusions. None occurred intraoperatively. Only two patients (0.12%) required red cell transfusions due to postoperative surgical bleeding (one following perforated appendicitis and one unexplained hemorrhage). The other three transfusions were for non-surgical causes: one patient with sickle cell disease (red cells), one with postoperative anemia (fresh frozen plasma), and one with sepsis-related coagulopathy (platelets). Our overall red cell transfusion rate (0.17%) did not differ significantly from published rates (range 0-0.46%; all p>0.05). There were no deaths. Routine G+S testing for all 1,733 patients would have cost approximately £26,000-£36,500 over the 10 years (≈£15-£21 per test); with only two clinically relevant surgical transfusions, the cost per such case would exceed £13,000.

Conclusion: Routine preoperative G+S testing before laparoscopic appendectomy provides minimal clinical benefit in low-risk patients and represents unnecessary resource use. Our 10-year data show an extremely low transfusion rate, with most transfusions driven by patients' underlying conditions rather than operative bleeding. These findings support a selective, risk-based approach to G+S: reserve testing for patients with significant comorbidities or anticipated complex surgery while omitting routine G+S in low-risk cases. Such a strategy would maintain patient safety but reduce laboratory workload and costs.

## Introduction

Acute appendicitis is among the most common surgical emergencies and accounts for roughly one-fifth of acute abdominal presentations [1]. Laparoscopic appendectomy is the standard of care because it is associated with lower morbidity, reduced pain, and faster recovery than open surgery [1]. Historically, many centres mandated routine preoperative group-and-save (G+S) testing for laparoscopic general surgery, including appendectomy, based on the premise that transfusion might occasionally be required; recent UK series specifically examining appendectomy have questioned this practice [2,3], and earlier work describes the rationale for routine preoperative screening in emergency laparoscopy [4].

Across contemporary series and a systematic review, perioperative transfusion after laparoscopic appendectomy is exceedingly rare, with reported rates between 0% and 0.46% [5-7]; even in complicated or perforated appendicitis, most patients do not require transfusion [6]. Improvements in technique, visualization, and hemostatic devices likely contribute to the very low bleeding risk in modern practice [1,5]. Despite these data, routine preoperative G+S remains embedded in local preoperative checklists at many hospitals [2-4].

Notably, there is no national recommendation supporting routine G+S for low‑risk laparoscopic procedures, and the National Institute for Health and Care Excellence (NICE) NG45 advises against blanket preoperative testing in such cases [8]. Each G+S sample consumes laboratory time (often close to an hour when performed urgently) and adds cost to pathways already under pressure [9,5]. Several authors therefore advocate a selective, risk‑based approach, reserving G+S for patients with significant comorbidity or anticipated complexity, rather than universal testing [5,10].

The objective of the present study was to evaluate, over a 10‑year period at a single UK centre, the clinical necessity and cost‑effectiveness of routine preoperative G+S in laparoscopic appendectomy, by quantifying transfusion rates, classifying indications, and estimating potential resource impact.

## Materials and methods

This retrospective observational study was conducted in the General Surgery Department at Homerton University Hospital (London, UK). Institutional audit approval was obtained (audit no. 22715). We identified all patients who underwent emergency or elective laparoscopic appendectomy between October 15, 2014, and October 15, 2024, using the electronic patient record system. Patients undergoing open appendectomy or concurrent abdominal/gynaecological procedures were excluded.

For each patient, we recorded demographic data (age, sex) and operative details. We identified all patients who received any blood transfusion during the perioperative period. For each transfused patient, operative notes and discharge summaries were reviewed by two independent investigators to establish the indication for transfusion (surgical bleeding vs. other causes) and timing (intraoperative vs. postoperative). All data extraction was performed independently by two reviewers and anonymized prior to analysis.

Descriptive statistics summarized the patient cohort and transfusion data. We compared our transfusion rate to those reported in six published studies of laparoscopic appendectomy or cholecystectomy using two-proportion z-tests (significance p<0.05). Our analysis focused on red cell transfusions attributable to surgical bleeding. We also performed a cost analysis: assuming a National Health Service (NHS) cost of £15-£21 per G+S test, we calculated the total cost of performing routine G+S on all patients over the study period.

## Results

**Figure 1:**
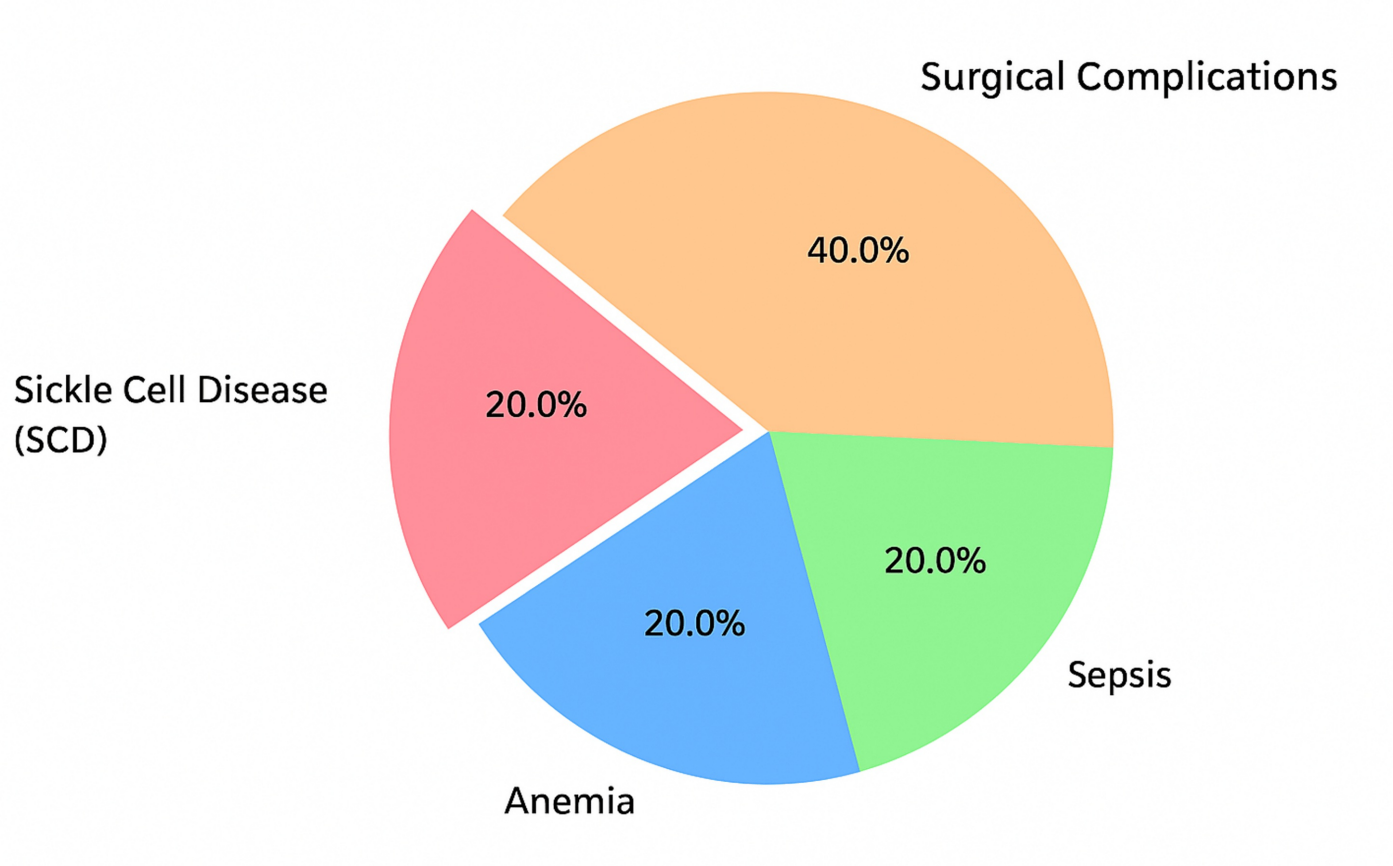
Causes of perioperative transfusion (n=5): surgical bleeding (n=2), sickle cell disease (n=1), postoperative anemia (n=1), and sepsis-related coagulopathy (n=1)

A total of 1,733 patients met the inclusion criteria. The mean age was 51 years (range 7-86), with 925 males (53%) and 808 females (47%). Overall, five patients (0.29%) required blood transfusion, and none of these occurred intraoperatively. Two patients required red cell transfusion for postoperative bleeding related to the surgery (one after perforated appendicitis and one for unexplained postoperative hemorrhage). The other three transfusions were unrelated to surgical bleeding: one patient with known sickle cell disease received postoperative red cells, one patient with postoperative anemia received fresh frozen plasma, and one patient with sepsis-related coagulopathy received platelets. In total, three patients (0.17%) received red cell transfusions, of which two (0.12%) were directly attributable to surgical bleeding (Figure 1). There were no transfusion-related deaths.

When compared with six published series (Table 1), reported transfusion rates ranged from 0% to 0.46%. Our red cell transfusion rate (0.17%) did not differ significantly from any of the reported rates (all p>0.05). This consistency with existing data reinforces the rarity of transfusion in laparoscopic appendectomy.

**Table 1:** Rate of transfusion in the literature compared to our data set

Study	Transfusions	Total cases	Transfusion rate (%)	Z-score	P-value
Our data	3	1,733	0.17	0.00	1.000
Usal et al., 1999 [11]	12	2,589	0.46	-1.59	0.1116
Ghirardo et al., 2010 [12]	11	3,424	0.32	-0.97	0.3341
Barrett-Lee et al., 2018 [4]	0	469	0.00	0.90	0.3672
Magowan et al., 2020 [6]	0	361	0.00	0.79	0.4289
Blank et al., 2018 [7]	2	751	0.27	-0.48	0.6341
Farrell et al., 2020 [13]	1	645	0.16	0.10	0.9238

Routine G+S testing of all patients (n=1,733) would have incurred approximately £26,000-£36,500 in laboratory costs over 10 years. Given that only two transfusions were needed for surgical bleeding, the effective cost per clinically relevant transfusion exceeds £13,000.

## Discussion

In this 10-year single-centre review of 1,733 laparoscopic appendectomy cases, transfusions were exceedingly rare. Only five patients (0.29%) required any blood products, and crucially, only two (0.12%) needed red cell transfusion due to operative bleeding, both identified postoperatively. The remaining transfusions were for non-surgical reasons (sickle cell disease, anemia, sepsis). Overall, the rate of red cell transfusion for surgical bleeding was 0.12% and 0.17% for any red cell transfusion. There were no intraoperative transfusions or perioperative deaths.

These results align with prior studies showing minimal need for transfusion in this setting. Several UK and international series (and systematic reviews) have reported similarly low or zero transfusion rates for laparoscopic appendectomy [2-4,9-13]. For example, Magowan et al. found a 0% transfusion rate in 361 laparoscopic appendectomies [6]. In our comparative analysis, our transfusion rate fell within the range reported in the literature and did not differ significantly (all p>0.05), reinforcing that significant hemorrhage is highly unlikely during routine laparoscopic appendectomy.

The traditional rationale for routine G+S, fear of catastrophic hemorrhage at laparoscopic entry, is now largely theoretical. Modern laparoscopic techniques and instrumentation (including open-entry or direct-vision entry methods) have greatly reduced the risk of major vascular injury. In our series, almost all transfusions were for pre-existing medical conditions rather than intraoperative bleeding, which supports a selective approach [14]. We recommend reserving G+S for patients with documented bleeding disorders, significant anemia, or anticipated complex surgery. In unexpected hemorrhage during emergency cases, the use of universal donor (O-negative) blood until crossmatched blood is available can be applied safely.

From a resource standpoint, routine G+S is costly. Each G+S sample consumes laboratory time and materials. By our estimate, testing all 1,733 patients costs roughly £26,000-£36,500 over 10 years (assuming £15-£21 per test). Other authors have highlighted similar potential savings by limiting G+S to those who truly need it [15-17]. The NICE guideline on preoperative testing (NG45, 2016) also explicitly advises against routine G+S for low-risk procedures [8]. Our data suggest that adopting a targeted, risk-based G+S policy would maintain patient safety while optimizing resource use and reducing unnecessary workload.

## Conclusions

Routine preoperative G+S testing prior to laparoscopic appendectomy provides minimal clinical benefit in low-risk patients. The incidence of transfusion for surgical bleeding is extremely low (0.12% in our series), and most transfusions occur due to patients' underlying conditions rather than the procedure itself. Consistent with national guidance and international literature, our findings support a more selective, risk-based approach to preoperative G+S testing. Limiting G+S to patients with significant comorbidities, abnormal preoperative hematology, or anticipated intraoperative complexity would maintain safety while substantially reducing costs and laboratory.
